# Software application profile: mrrobust—a tool for performing two-sample summary Mendelian randomization analyses

**DOI:** 10.1093/ije/dyy195

**Published:** 2018-09-12

**Authors:** Wes Spiller, Neil M Davies, Tom M Palmer

**Affiliations:** 1 Population Health Science Institute, University of Bristol, Bristol, UK; 2 Department of Mathematics and Statistics, Lancaster University, Lancaster, UK

**Keywords:** Summary MR, MR-Egger, IVW, weighted median, Mendelian randomization, Stata

## Abstract

**Motivation:**

In recent years, Mendelian randomization analysis using summary data from genome-wide association studies has become a popular approach for investigating causal relationships in epidemiology. The mrrobust Stata package implements several of the recently developed methods.

**Implementation:**

mrrobust is freely available as a Stata package.

**General features:**

The package includes inverse variance weighted estimation, as well as a range of median, modal and MR-Egger estimation methods. Using mrrobust, plots can be constructed visualizing each estimate either individually or simultaneously. The package also provides statistics such as$\;I_{GX}^{2}$, which are useful in assessing attenuation bias in causal estimates.

**Availability:**

The software is freely available from GitHub [https://raw.github.com/remlapmot/mrrobust/master/].

**Key Messages**

- The mrrobust software package facilitates two-sample summary MR analyses using summary data from genome-wide association studies.
- The package allows for implementation of a range of summary MR estimators using Stata, improving the extent to which results are reproducible.
- Conclusions from the supported analyses can be robust to sources of confounding bias and pleiotropy, though findings should be considered with respect to the underlying assumptions of each estimator.

## Introduction

Mendelian randomization^1^ has developed into a popular approach to examining causal relationships in epidemiology.^2^^,^^3^ By employing genetic variants as instrumental variables (IVs) it is possible to limit bias from confounding, provided variants satisfy the assumptions of IV analysis.^1^^,^^4^ For a genetic variant to serve as a suitable instrument, three assumptions must hold: (i) it must be associated with the exposure of interest; (ii) there must be no confounders of the instrument and outcome; and (iii) the instrument must not affect the outcome except via the exposure of interest.^5^

Candidate variants are usually identified through large genome-wide association studies (GWASs).^6^ However, IV analyses using single variants rarely have sufficient power to test hypotheses of interest.^6^^,^^7^ One approach to increase the statistical power of Mendelian randomization studies is to use multiple genetic variants as instruments within a two-sample summary framework.^8^^,^^9^ Two-sample Mendelian randomization estimates the effect of the exposure using instrument-exposure and instrument-outcome associations from different samples, often through methods originally developed for meta-analysis.^8^^,^^9^ This is particularly useful as MR estimators, such as MR Egger and median based regression, are robust to certain forms of violation of the third instrumental variable assumption.^8^^,^^10^^,^^11^ Violations of this assumption can occur through directional pleiotropy, where a genetic variant affects the study outcome through pathways that are not mediated via the exposure. Such developments have contributed to the increasing popularity of two-sample summary MR.^5^

This paper introduces the mrrobust Stata package as a tool to help researchers implement two-sample MR analyses, and can be viewed as the Stata counterpart to toolkits such as the MR-Base web application, and the MendelianRandomization and TwoSampleMR R packages.^12^^,^^13^ Whereas it is possible to conduct individual-level IV analyses in Stata using modules such as IVREG2,^14^ two-sample summary MR has previously required bespoke code to implement. The mrrobust package addresses this limitation, providing a suite of popular two-sample MR methods and sensitivity analyses. Before continuing, we briefly outline the three primary estimation methods included in the mrrobust package, using the notation of Bowden *et al.*^10^^,^^15^

## Methods

### Inverse variance weighting (IVW)

To perform IVW, a weighted average$\;{\hat{\beta }}_{IVW}$ is calculated using the set of ratio estimates ${\hat{\beta }}_{J}$ for each individual variant J=1, 2,…,j.^9^ Ratio estimates are obtained for each variant by dividing the instrument-outcome association by the corresponding instrument-exposure association. Such association estimates are obtained by fitting simple linear regression models of the outcome and exposure upon the genetic variant, primarily by conducting a GWAS. Let$\;{\hat{\gamma }}_{j}$ and $\sigma_{Yj}^{2}$ denote the instrument-outcome association and variance, respectively, for the$\;j^{th}$ variant. The IVW estimate is then defined as:

$${\hat{\beta }}_{IVW}=\frac{\sum_{j=1}^{J}w_{j}{\hat{\beta }}_{j}}{\sum_{j=1}^{J}w_{j}},\;w_{j}=\frac{{\hat{\gamma }}_{j}^{2}}{\sigma_{Yj}^{2}}$$

This corresponds to the estimate one would obtain from a weighted linear regression of the set of instrument-outcome associations upon the set of instrument-exposure associations, constraining the intercept at the origin.^9^ One drawback of the IVW approach is that causal effect estimates can be biased in cases where one or more variants exhibit directional pleiotropy.^9^

### MR-Egger regression

MR-Egger regression is valid under weaker assumptions than IVW, as it can provide unbiased causal effect estimates even if the variants have pleiotropic effects. In this case, the set of instrument-outcome associations is regressed upon the set of instrument-exposure associations, weighting the regression using precision of the instrument-outcome associations, as in the IVW case.^8^ However, MR-Egger does not constrain the intercept at the origin, and the intercept represents an estimate of the average directional pleiotropic effect across the set of variants. The slope of the model provides an unbiased estimate of the causal effect.^8^^,^^10^ If there is little evidence of systematic differences between the IVW and MR-Egger, then the IVW should be preferred. The IVW is more efficient, but potentially less robust, and in such cases the IVW estimate is often the most appropriate estimate to adopt due to the greater precision of IVW estimates in comparison with other approaches.^10^ If there are differences between the IVW and MR-Egger estimates, this may be due to pleiotropy or to heterogeneous treatment effects.

The utility of MR Egger regression hinges upon two core assumptions. First, the INstrument Strength Independent of Direct Effect (InSIDE) assumption requires the effects of single nucleotide polymorphisms (SNPs) on the exposure and their pleiotropic effects on the outcome to be independent. If the InSIDE assumption holds, estimates for variants with stronger instrument-exposure associations$\;(\hat{\gamma_{j}})$ will be closer to the true causal effect parameter than variants with weaker associations.^8^ Second, the NO Measurement Error (NOME) assumption requires no measurement error to be present in the instrument-exposure associations, and therefore that the variance of the instrument-exposure association$\;\sigma_{Xj}^{2}=0$. In cases where NOME is strictly satisfied, estimates$\;{\hat{\gamma }}_{j}$ will be equal to$\;\gamma_{j}$ and the variance of the ratio estimate for each variant j is $var\left({\hat{\beta }}_{j}\right)=\frac{\sigma_{Yj}^{2}}{{\hat{\gamma }}_{j}^{2}}$. We further note that the NOME assumption applies to other two-sample MR approaches and is not therefore a unique feature of the MR Egger approach.

In cases where the NOME assumption is violated, individual variants will suffer from weak instrument bias, leading to attenuation of MR Egger estimates towards the null. This can occur if the SNPs were not genome-wide significant ($p=5\times 10^{-8}$) or were selected from small GWAS. One novel approach to assessing the strength of the NOME assumption is to evaluate the$\;I_{GX}^{2}$ statistic, interpreted as the relative degree of attenuation bias in the MR Egger regression in the interval (0, 1).^10^ Thus, for example, an $I_{GX}^{2}$ value of 0.7 represents an estimated relative bias of 30% towards the null. Further details regarding calculation of the $I_{GX}^{2}$ statistic are presented in the Supplementary material, available at *IJE* online.

### Weighted median

The weighted median approach is an adaptation of the simple median estimator for two-sample summary MR.^15^ For a total number of variants J=2k+1, the simple median approach selects the middle ratio estimate$\;{\hat{\beta }}_{k+1}$, from ordered ratio estimates$\;{{\hat{\beta }}_{1},{\hat{\beta }}_{2},\ldots \hat{\beta }}_{j}.$^15^ In cases where the total number of variants is even, the median is interpolated as$\;\frac{1}{2}\left({\hat{\beta }}_{k}+{\hat{\beta }}_{k+1}\right)$. As the simple median approach is inefficient, particularly in cases with variable precision in the set of ratio estimates, it is preferable to incorporate weights in a fashion similar to the IVW and MR Egger approaches. Let$\;s_{j}=\sum_{k=1}^{j}w_{k}$ be the sum of weights for the set of variants 1, 2,…j, standardized so the sum of weights$\;s_{J}$=1. The weighted median estimator is the median of a distribution having estimate ${\hat{\beta }}_{j}$ as its$\;p_{j}=100{\left(s_{j}-\frac{w_{j}}{2}\right)}^{th}$ percentile.^15^ For the range of percentile values, we perform a linear extrapolation between neighbouring ratio estimates.

An important assumption of the median summary MR approaches is that more than 50% of the genetic variants do not exhibit directional pleiotropy. In the simple median case, this threshold refers to the number of variants, whereas in the weighted median case, the 50% threshold is with respect to the weights of the non-pleiotropic variants.^15^

### Additional estimators

As two-sample MR represents a developing area of genetic epidemiology, novel approaches to causal effect estimation are incorporated into the mrrobust package through frequent updates. One such method is the mode-based estimator put forward by Hartwig *et al.*^16^ Details on the implementation of this approach with accompanying examples can be found in the Supplementary material, available at *IJE* online.

### Visualizing MR estimates

One useful approach to presenting the results of MR analyses is to produce a scatterplot, with the x and y axes representing the instrument-exposure and instrument-outcome associations, respectively, for each variant. If one were to draw a hypothetical regression line leading from the origin to each variant, the slope of the line would represent a ratio estimate of the causal effect using the single variant as an instrument, that is dividing the instrument-outcome association by the instrument-exposure association (defined as $\beta_{j}$ above). The precision of the instrument-outcome association estimate for each variant is illustrated using vertical error bars, whereas horizontal error bars pertaining to the instrument-exposure association may be omitted for clarity. As the IVW, MR-Egger, median and modal approaches essentially meta-analyse the set of ratio estimates, it is possible to include regression lines highlighting effect estimates of each approach for comparison. For such regression lines, positive and negative slopes are indicative of a positive or negative effect, respectively, whereas a slope of zero represents the absence of an observed association.

## Implementation

The mrrobust package uses functions from moremata,^17^ addplot^18^ and the heterogi^19^ command. For versions of Stata 13 and higher, it can be installed using the .net install command from [https://raw.github.com/remlapmot/mrrobust/master/]. For older versions of Stata, a zip archive of the files is freely available for download at: [https://github.com/remlapmot/mrrobust].

The package facilitates two-sample summary MR analyses with key features including:

- IVW and MR-Egger regression approaches, including fixed effects MR-Egger regression, standard error correction and weighting options;
- unweighted, weighted and penalized weighted median IV estimators, providing pleiotropy robust estimates in cases where fewer than 50% of the genetic instruments are valid;
- modal estimation following Hartwig *et al.*,^16^ including weighted and unweighted variations;
- presentation of heterogeneity statistics, statistics such as $I_{GX}^{2}$ for use in assessing attenuation bias,^10^ and Simulation Extrapolation (SIMEX) correction following Bowden *et al*;^10^
- plotting tools to visualize IVW, MR-Egger and weighted median estimators, as well as density plotting with respect to implementing the modal estimator;
- and illustrative examples and documentation using data from Do *et al.*^20^

### Applied examples: adiposity and height as predictors of serum glucose levels

To illustrate key features of the mrrobust package, we perform two analyses investigating potential relationships between adiposity, height and serum glucose. Adiposity was selected owing to the vast body of evidence supporting a positive association with serum glucose levels,^21–24^ whereas height was based upon limited evidence of association.^25–27^ Glucose was selected as an outcome with respect to its hypothesized role in the development of type 2 diabetes.^21^^,^^27^ Datasets were obtained from the MR-Base web application and pruned for linkage disequilibrium before conducting the analyses.^13^

### Applied example I: adiposity and serum glucose

Though the relationship between adiposity and glucose has received much attention in the literature, such studies are predominantly observational and therefore may be subject to bias from confounding. This provides motivation for considering Mendelian randomization techniques which are able to control for such unobserved confounding. In the initial analysis, we select adiposity as an exposure measured using standardized body mass index (BMI), obtaining estimates of its associations with genotypes and their respective standard errors from Locke *et al.*^28^

For the outcome, we consider log transformed measures of serum glucose log(mM) using effect estimates and standard errors from Shin *et al.*^29^ The summary data used for this analysis are provided in the Supplementary material, available at *IJE* online. Adopting a GWAS significance *P*-value threshold of $5\times 10^{-8},$ a total of 79 independent SNPs were identified in both samples. We confirmed the linkage equilibrium (LD) between the SNPs using a clumping algorithm, a clumping distance of 10000 kb and an LD $R^{2}$ of 0.001. This resulted in a total of 79 SNPs for use as instrumental variables, details of which are presented in the Supplementary material, available at *IJE* online.

Using mrrobust, we conducted IVW, MR-Egger and weighted median regression approaches using the above summary data. The code for our analysis is in the Supplementary material, available at *IJE* online. For IVW and MR Egger approaches, the regression was weighted using the variance of the instrument-outcome association. The set of summary MR estimates are presented in Table 1A.

**Table 1.** Summary MR estimates for the effect of standardized BMI (A) and height (B) upon log transformed serum glucose

	Estimate	SE	*P*-value	95% CI
**BMI (A)**				
IVW				
Effect	0.023	0.008	0.004	0.01, 0.04
MR Egger				
Intercept	0.000	0.001	0.948	−0.001, 0.001
Effect	0.022	0.022	0.325	−0.02, 0.07
Weighted median				
Effect	0.034	0.012	0.005	0.01, 0.06
**Height (B)**				
IVW				
Effect	0.002	0.003	0.641	−0.005, 0.008
MR Egger				
Intercept	0.0001	0.0003	0.627	−0.0001, 0.0001
Effect	0.003	0.009	0.777	−0.02, 0.02
Weighted median				
Effect	<0.0001	0.005	>0.99	−0.01, 0.01

SE, standard error.

**Figure 1.**
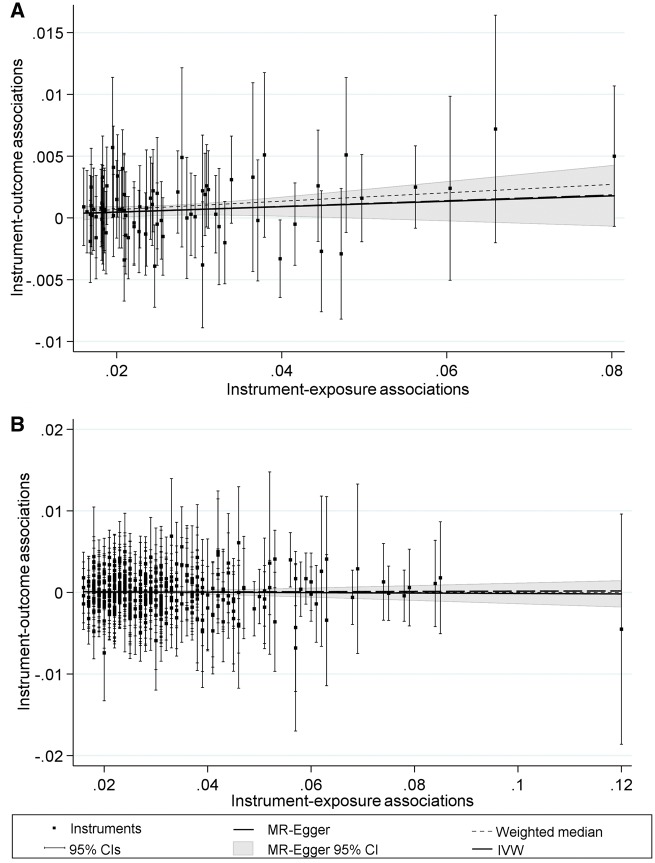
mreggerplot output for applied examples using BMI (A) and height (B) as exposures.

We find strong evidence of a positive association between BMI and serum glucose, using both IVW and weighted median methods. Considering the MR Egger case, a substantial average directional pleiotropic effect was not detected, and the lack of significance with respect to the effect estimate can be attributed to a lack of statistical power. An$\;I_{GX}^{2}$ value of 0.88 was reported, which can be interpreted as a relative bias in the MR-Egger estimate of 12% towards the null. The estimates are shown in Figure 1A, constructed using the mreggerplot command which generates a scatterplot of the instrument-exposure and instrument-outcome associations for each variant. This shows the set of estimates to be in agreement, with the plot being constructed as previously described.

### Applied example II: height and serum glucose

As a further example, we consider the effect of standardized height (metres) upon serum glucose, using summary data from Wood *et al*.^30^ and outcome summary data on log transformed serum glucose from Shin *et al.*^29^ The summary data used for this analysis are provided in the Supplementary material, available at *IJE* online. We assess the SNPS for LD using criteria from the previous example and identify 367 SNPs as suitable instruments for the analysis, details of which are presented in the Supplementary material as above. The set of summary MR estimates are presented in Table 1B.

From Table 1B, we find no evidence against the null hypothesis of no association between height and serum glucose levels using IVW, weighted median and MR Egger regression. Considering the MR Egger case, there appeared to be no evidence of directional pleiotropy, with an $I_{GX}^{2}$ value of 0.90 indicating a relative bias of 10% towards the null. As in the previous example, a plot of the MR estimates can be generated using the mreggerplot command as shown in Figure 1B. In this scenario, the estimates appear in agreement, indicating a lack of evidence for a substantial directional pleiotropic effect.

## Discussion

The mrrobust package is a freely available Stata package, containing a number of summary MR estimation methods which can be used to estimate causal effects. In the applied example, the mrrobust package was able to provide a series of estimates, finding evidence of a positive association between BMI and serum glucose and no evidence of association between height and serum glucose. One possible conclusion that can be drawn from these results is that previously reported associations between height and glucose are driven by confounding factors.^31^^,^^32^ It is important, however, to consider the extent to which Mendelian randomization is appropriate for a given analysis and, by extension, situations in which mrrobust is suitable.

In the first instance, Mendelian randomization studies only produce unbiased estimates when genetic instruments satisfy the assumptions of each estimator (e.g. IVW, MR-Egger or weighted median). In two-sample analyses, genetic instruments should be associated with the exposure of interest at genome-wide levels of significance (satisfying the first instrumental variable assumption), and pruned for LD to limit the overlap between SNPs. The IVW estimator also requires that genetic variants should not have directional pleiotropic effects. The MR Egger and median estimators are robust to directional pleiotropy if the effects of the exposure are constant. MR Egger regression requires the InSIDE assumption, whereas median methods assume that the number of valid instruments is greater than 50%. For MR-Egger estimation where the value of $I_{GX}^{2}$ is low, it is possible to use SIMEX to correct for regression attenuation towards the null. This is implemented using the mreggersimex command.

In this paper, we have presented the mrrobust Stata package as an accessible toolkit for performing summary MR and instrumental variable analysis using many instruments. It contains a range of summary MR approaches, and should make examining causal relationships using Mendelian randomization more accessible for genetic epidemiologists.

## Funding

The Medical Research Council (MRC) and the University of Bristol fund the MRC Integrative Epidemiology Unit (MC UU 12013/1, MC UU 12013/9, MC UU 00011/1). W.S. is funded by the Wellcome Trust (108902/B/15/Z).

## Supplementary Material

dyy195_Supplementary_Material
