# Supplementary material for: Software application profile: mrrobust—a tool for performing two-sample summary Mendelian randomization analyses
Source: Int J Epidemiol. 2018 Sep 12;48(3):684–90. doi: 10.1093/ije/dyy195 (PMC6659377; doi:10.1093/ije/dyy195)
Supplement: dyy195_Supplementary_Material [file dyy195_supplementary_material.zip › dyy195-suppl_data/ije-2017-05-0618-File003.docx]

Supplementary Material (updated 17/07/18)

Within this supplementary document we include example code and Stata output for each of the analyses performed within this paper. We also include a brief summary of the $I_{GX}^{2}$ statistic, as well as guidance on implementing and interpreting the modal estimator. The summary data used in the applied analyses are described below and contained within the two comma delimited files ***BMI.csv*** and ***Height.csv*** which accompany this document. These represent summary data estimates for the BMI-serum glucose and height-serum glucose analyses respectively.

Contents

[Stata code for implementing two-sample summary MR 2](#_Toc518655230)

[Stata output for each estimation method using mrrobust: BMI-Serum Glucose 3](#_Toc518655231)

[Stata output for each estimation method using mrrobust: Height-Serum Glucose 4](#_Toc518655232)

[Description and calculation of the $IGX2$ statistic 5](#_Toc518655233)

[Implementation of the mode-based estimator 6](#_Toc518655234)

[Stata output using the mode-based estimator using mrrobust: BMI-Serum Glucose 9](#_Toc518655235)

[Stata output using the mode-based estimator using mrrobust: Height-Serum Glucose 11](#_Toc518655236)

[References 12](#_Toc518655237)

# Summary data description and overview

Accompanying this paper are two sets of data ***BMI.csv***, and ***Height.csv***, containing the set of summary estimates required for performing the BMI-serum glucose and height-serum glucose analyses respectively. Each dataset is organised into 5 columns under the following headings:

- SNP: A set of identifying numbers (rsids) for each genetic variant
- beta.exposure: a set of values representing the coefficient from regressing the exposure upon the genetic variant within a GWAS
- beta.outcome: a set of values representing the coefficient from regressing the outcome upon the genetic variant within a GWAS
- se.exposure: a set of values representing the standard error corresponding to the coefficient in beta.exposure
- se.outcome: a set of values representing the standard error corresponding to the coefficient in beta.outcome

In ***BMI.csv*** the exposure is standardised body mass index (BMI), and is therefore interpreted on a standard deviation scale. The summary statistics are reported by Locke et al(1).

In ***Height.csv*** the exposure is standardised height in meters and also interpreted on a standard deviation scale. The summary statistics are reported by Wood et al(2).

For both analyses log transformed serum glucose was used as an outcome, reported by Shin et al(3). All the data was obtained from the MRBase GWAS catalogue available at <http://www.mrbase.org/> (4). Genetic variants were pruned so as to be independent ($R^{2}=0.0001$), and the effect alleles were aligned between the exposure and outcome datasets using the MRBase web application, prior to implementing mrrobust.

# Stata code for implementing two-sample summary MR

**betaoutcome: variable of gene-outcome associations**

**betaexposure: variable of gene-exposure associations**

**seoutcome: variable of gene-outcome association standard errors**

**seexposure: variable of gene-exposure association standard errors**

**############################IVW estimation#############################**

**//Perform IVW for effect of exposure upon outcome**

**mregger betaoutcome betaexposure [aw=1/(seoutcome^2)], ivw**

**##################Fixed-Effects MR-Egger estimation####################**

**//Perform random effects MR-Egger for effect of exposure upon outcome**

**mregger betaoutcome betaexposure [aw=1/(seoutcome^2)]**

**####################Weighted Median estimation#########################**

**//Perform weighted median for effect of exposure upon outcome**

**mrmedian betaoutcome seoutcome betaexposure seexposure, weighted**

**################Generate Plot showing summary MR estimates#############**

**//Produce combined plot showing IVW, MR-Egger and weighted median estimates**

**mreggerplot betaoutcome seoutcome betaexposure seexposure**

**#########Perform Modal Estimation default bandwidth values and#########**

**###################construct overlaid density plots####################**

**//Produce estimates and density plots for modal estimation**

**mrmodalplot betaoutcome seoutcome betaexposure seexposure, lc(gs10 gs5 gs0)**

# Stata output for each estimation method using mrrobust: BMI-Serum Glucose

**IVW**

**
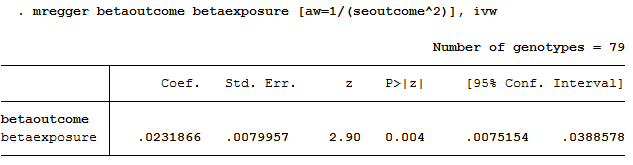
**

**MR-Egger**

**
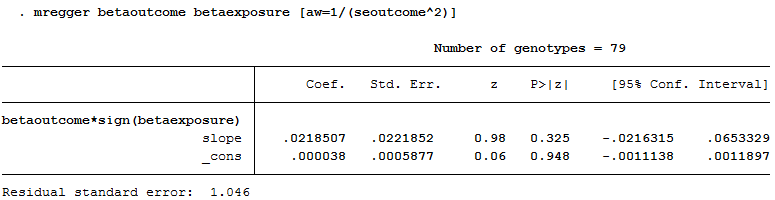
**

**Weighted Median**

**
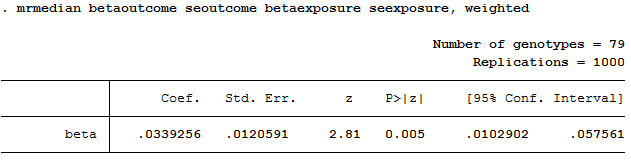
**

# Stata output for each estimation method using mrrobust: Height-Serum Glucose

**IVW**

**
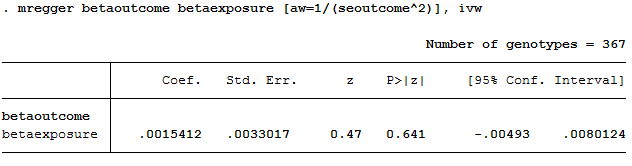
**

**MR-Egger**

**
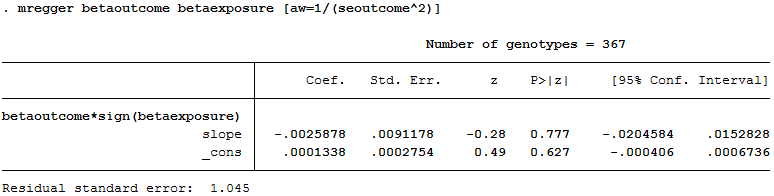
**

**Weighted Median**

**
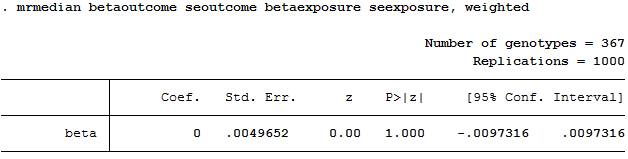
**

# Description and calculation of the $I_{GX}^{2}$ statistic

The $I_{GX}^{2}$ statistic, as described in Bowden et al (2016)(5), is an adaptation of the conventional $I^{2}$ heterogeneity measure used in meta-analysis. The $I_{GX}^{2}$ statistic estimates the expected degree of regression dilution resulting from violation of the NOME assumption, providing a value within the interval of 0 and 1 interpreted as the relative bias of the two-sample MR Egger estimate towards the null. For example, a value $I_{GX}^{2}=0.9$ would be interpreted as 10% relative bias towards the null.

To calculate $I_{GX}^{2}$ it is first necessary to define Cochran’s Q statistic for the range of instrument-exposure associations. Adopting notation from Bowden et al(5), for a set of genetic instruments $L=\left\{ 1,2,\ldots j \right\}$:

$$Q_{GX}=\sum_{j=1}^{L} \frac{\left( \hat{\gamma}_{j}-\bar{\hat{\gamma}} \right)^{2}}{\sigma_{Xj}^{2}}$$

Where $\hat{\gamma}_{j}$ is the instrument-exposure association for the $j^{th}$ genetic instrument, and $\sigma_{Xj}^{2}$ is corresponding precision of the instrument-exposure association for the $j^{th}$ genetic instrument used as a weight. It is then possible to calculate $I_{GX}^{2}$ as

$$I_{GX}^{2}=\left( Q_{GX}-\left( L-1 \right) \right)/Q_{GX}$$

# Implementation of the mode-based estimator

The mode-based estimator assumes that the model ratio estimate will be a consistent estimator of the causal effect, encapsulated within the ZEro Modal Pleiotropy Assumption (ZEMPA). It follows a similar intuitive framework to median based approaches, with two key advantages:

1. The ZEMPA assumption is weaker than assuming more than 50% of the variants are valid.
2. The ZEMPA assumption can be visually assessed by constructing a density plot using the set of individual variant ratio estimates.

As a starting point, it is useful to consider density plots using the set of ratio estimates for each genetic variant. Such density plots should initially be constructed using several bandwidth values (denoted $\phi$) to aid in assessing plausibility of the ZEMPA assumption. In ideal cases, a single discernible peak reduces ambiguity in selecting the modal value, whilst many peaks of similar height limit the extent to which a single modal value can be adopted. This is illustrated in Figure A1.


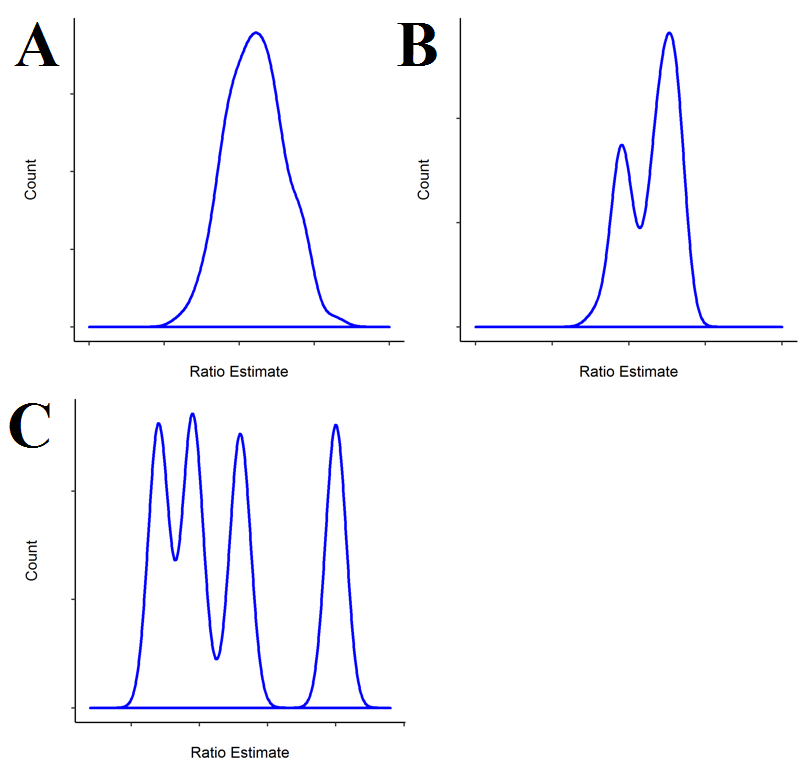


***Figure A1***: Several density plots illustrating ideal, reasonable, and problematic density plots with respect to applying the modal estimator.

In Figure A1, plot A represents the ideal case in which there is one clear peak. In plot B, there are two candidates for the modal value, however, in cases where there is a clear difference in density between the two peak values, one would select the greatest peak as the modal estimate. Finally, in plot C there appears be no justifiable way to select a single peak, and as such the modal estimator would not be appropriate to implement. A straightforward approach to applying the modal estimator is therefore to construct several density plots at different values of $\phi$, identify candidates for the modal ratio estimate, and fit a model of similar form to median based approaches, substituting the median estimate for the modal estimate. Note that standard errors are calculated by applying a parametric bootstrap, in a similar fashion to the median estimation.

Plot B also highlights further advantage of the mode-based estimator. In cases where two peaks are present at different ratio estimates, one of the peaks may be representative of a shared pleiotropic pathway between several genetic variants. In identifying such groups, researchers can look at shared phenotypic effects amongst the variants, and potentially uncover avenues of pleiotropic effects. As with the median approach, weighted and unweighted variations of the mode-based estimator are available. For further details see Hartwig et al(6).

# Stata output using the mode-based estimator using mrrobust: BMI-Serum Glucose

#### Using the mrmodalplot command, modal estimates are calculated using bandwidths of 0.25, 0.5, and 1 respectively. This command also produces three overlaid density plots for each value, as shown in Figure A2.

**
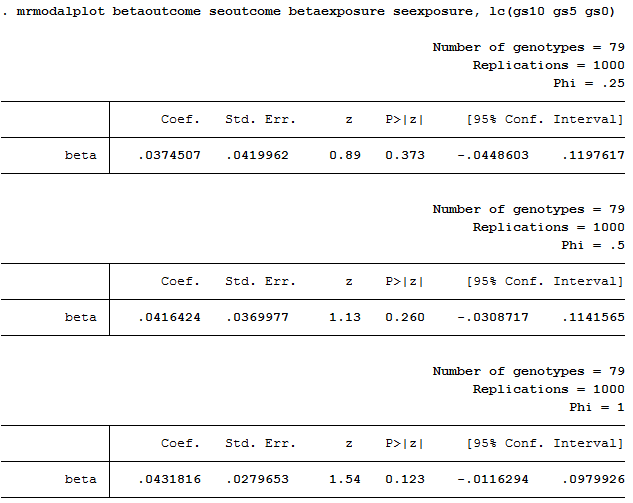
**


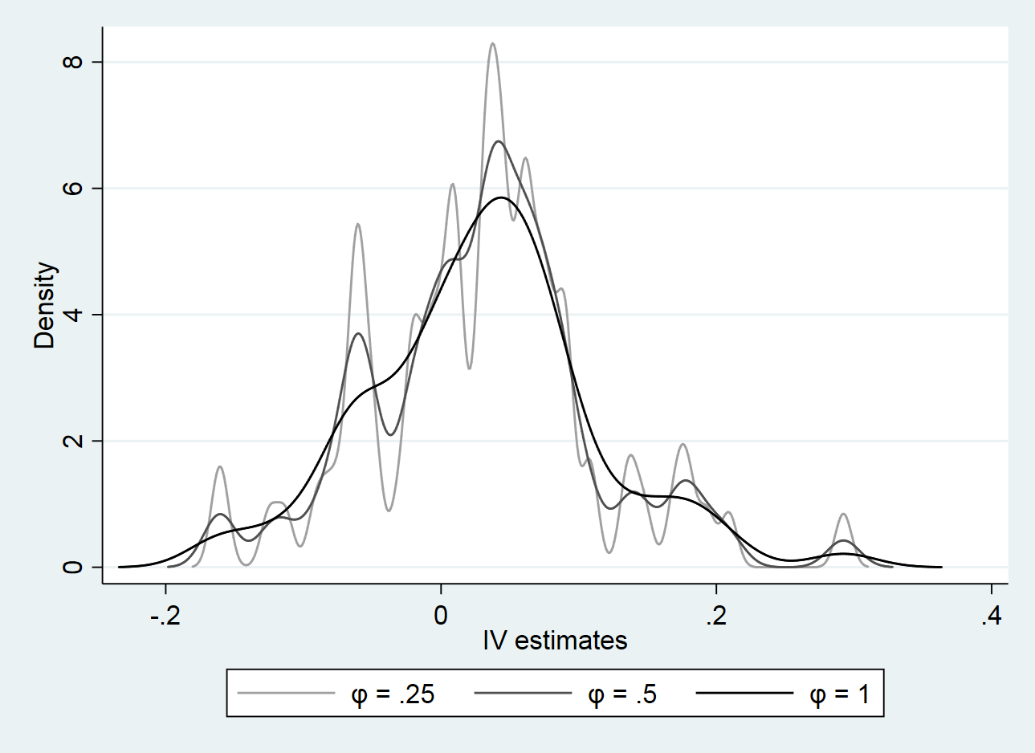


***Figure A2***: Output of the mrmodalplot command, showing density plots for the BMI-Serum Glucose data at bandwidths of 0.25, 0.5, and 1.

In this case, there appears to be a clear peak for at each value, though there may be some degree of ambiguity with respect to the tallest peaks using a bandwidth of 0.25. However, regardless of which bandwidth is adopted, the results appear in agreement with those of the alternative approaches previously considered.

# Stata output using the mode-based estimator using mrrobust: Height-Serum Glucose

As in the previous example, the mrrmodalplot command is used to fit the mode-based estimator at bandwidths of 0.25, 0.5, and 1. The Stata output is presented below, with density plots given in Figure A3.


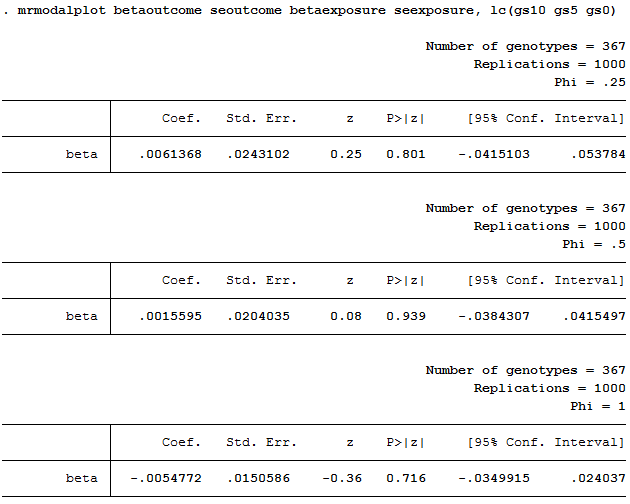


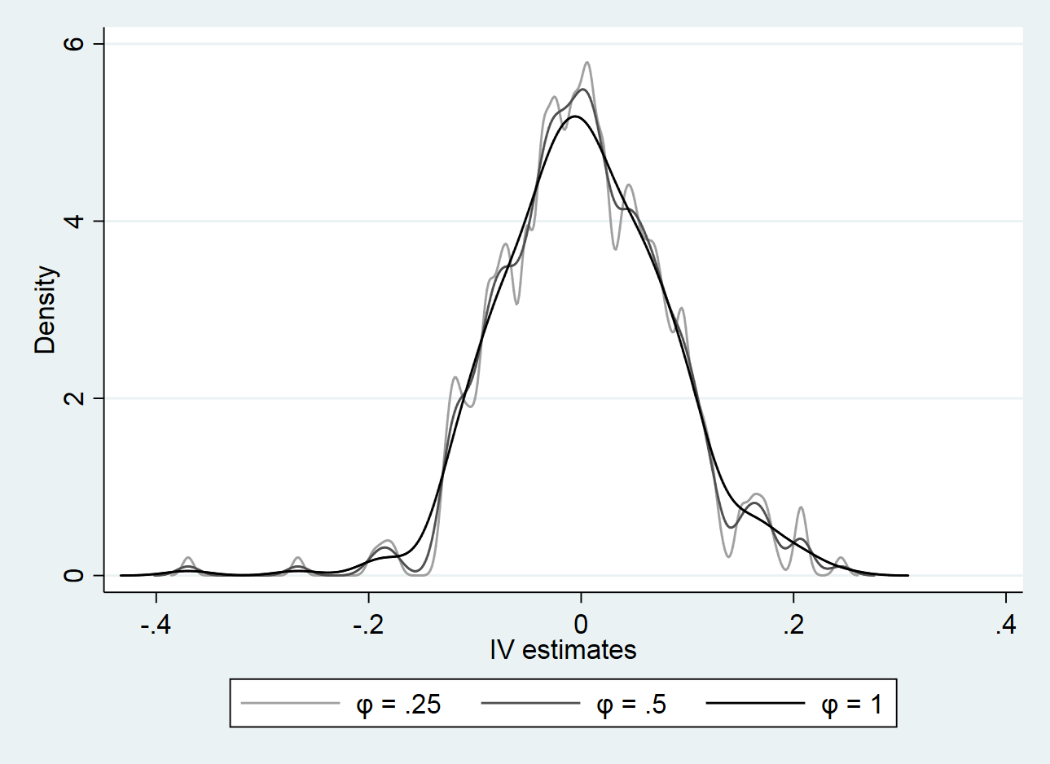


***Figure A3***: Output of the mrmodalplot command, showing density plots for the Height-Serum Glucose data at bandwidths of 0.25, 0.5, and 1.

# References

1. Locke AE, Kahali B, Berndt SI, Justice AE, Pers TH, Day FR, et al. Genetic studies of body mass index yield new insights for obesity biology. Nature. 2015;518(7538):197-206.

2. Wood AR, Esko T, Yang J, Vedantam S, Pers TH, Gustafsson S, et al. Defining the role of common variation in the genomic and biological architecture of adult human height. Nat Genet. 2014;46(11):1173-86.

3. Shin S-Y, Fauman EB, Petersen A-K, Krumsiek J, Santos R, Huang J, et al. An atlas of genetic influences on human blood metabolites. Nat Genet. 2014;46(6):543-50.

4. Hemani G, Zheng J, Elsworth B, Wade KH, Haberland V, Baird D, et al. The MR-Base platform supports systematic causal inference across the human phenome. Elife. 2018;7.

5. Bowden J, Del Greco MF, Minelli C, Davey Smith G, Sheehan NA, Thompson JR. Assessing the suitability of summary data for two-sample Mendelian randomization analyses using MR-Egger regression: the role of the I2 statistic. Int J Epidemiol. 2016;45(6):1961-74.

6. Hartwig FP, Davey Smith G, Bowden J. Robust inference in summary data Mendelian randomization via the zero modal pleiotropy assumption. Int J Epidemiol. 2017;46(6):1985-98.
